# Enhanced sensitivity to plasma p-tau217 with proper names recall in the preclinical Alzheimer’s cognitive composite

**DOI:** 10.1093/braincomms/fcag163

**Published:** 2026-05-06

**Authors:** Deling He, Rebecca Langhough, Erin Jonaitis, Rachael Wilson, Bruce Hermann, Henrik Zetterberg, Sterling Johnson, Kimberly D Mueller

**Affiliations:** Department of Communication Sciences and Disorders, University of Wisconsin-Madison, Madison, WI 53705, USA; Wisconsin Alzheimer's Disease Research Center, University of Wisconsin–Madison School of Medicine and Public Health, Madison, WI 53792, USA; Wisconsin Alzheimer’s Institute, University of Wisconsin—Madison School of Medicine and Public Health, Madison, WI 53726, USA; School of Nursing, University of Wisconsin-Madison, Madison, WI 53705, USA; Wisconsin Alzheimer's Disease Research Center, University of Wisconsin–Madison School of Medicine and Public Health, Madison, WI 53792, USA; Wisconsin Alzheimer’s Institute, University of Wisconsin—Madison School of Medicine and Public Health, Madison, WI 53726, USA; Department of Neurology, School of Medicine and Public Health, University of Wisconsin-Madison, Madison, WI 53705, USA; Wisconsin Alzheimer's Disease Research Center, University of Wisconsin–Madison School of Medicine and Public Health, Madison, WI 53792, USA; Department of Psychiatry and Neurochemistry, Institute of Neuroscience and Physiology, the Sahlgrenska Academy at the University of Gothenburg, Mölndal SE-413 45, Sweden; Clinical Neurochemistry Laboratory, Sahlgrenska University Hospital, Mölndal SE-431 80, Sweden; Department of Pathology and Laboratory Medicine, University of Wisconsin School of Medicine and Public Health, Madison, WI 53705-2281, USA; Department of Neurodegenerative Disease, UCL Institute of Neurology, Queen Square, London WC1N 3BG, UK; UK Dementia Research Institute at UCL, London NW1 3BT, UK; Centre for Brain Research, Indian Institute of Science, Bangalore 560012, India; Wisconsin Alzheimer's Disease Research Center, University of Wisconsin–Madison School of Medicine and Public Health, Madison, WI 53792, USA; Wisconsin Alzheimer’s Institute, University of Wisconsin—Madison School of Medicine and Public Health, Madison, WI 53726, USA; Department of Communication Sciences and Disorders, University of Wisconsin-Madison, Madison, WI 53705, USA; Wisconsin Alzheimer's Disease Research Center, University of Wisconsin–Madison School of Medicine and Public Health, Madison, WI 53792, USA; Wisconsin Alzheimer’s Institute, University of Wisconsin—Madison School of Medicine and Public Health, Madison, WI 53726, USA

**Keywords:** delayed recall, language biomarker, longitudinal decline, Alzheimer’s disease, logical memory

## Abstract

A pivotal debate in Alzheimer’s disease (AD) revolves around the clinical utility of a purely biomarker-based diagnostic framework, making it imperative to identify early functional signs that are both clinically meaningful and sensitive to biological biomarkers in the preclinical stages. While the development of the Preclinical Alzheimer’s Cognitive Composite (PACC) addresses this need, its lack of language-specific markers might limit its sensitivity, given that language deficits are among the earliest manifestations of AD. To address this limitation, we developed PACC variants incorporating proper names delayed recall, a novel lexical-semantic marker robustly linked to PET and CSF biomarkers. We analysed 824 dementia-free participants (mean age ≈ 62 at baseline) from the Wisconsin Registry for Alzheimer’s Prevention, stratifying them by plasma phosphorylated tau 217 (p-tau217) levels (using established amyloid PET positivity cut-offs: negative [<0.40 pg/mL; *N* = 539], intermediate [0.40–0.63 pg/mL; *N* = 163], positive [>0.63 pg/mL; *N* = 122]). We constructed six PACC variants: (1) traditional PACC4 (Rey Auditory Verbal Learning Test immediate learning, Logical Memory II [LM II; delayed recall], Digit Symbol, Mini-Mental State Examination [MMSE]); (2) PACC3 (excluding MMSE to mitigate ceiling effects); (3–4) PACC4_PN and PACC3_PN, replacing LM II’s total score with proper-name recall; and (5–6) PACC4 + VF and PACC3 + VF, adding animal fluency. Cognitive composites were calculated as the equally weighted sum of component z-scores. We compared PACC variants′sensitivity (i.e. longitudinal decline rates) associated with plasma p-tau217, using linear mixed models adjusted for age, gender, literacy and practice (total visits minus one). We showed individuals with positive plasma p-tau217 status exhibited significantly faster cognitive decline across all PACCs. Notably, the proper name enhanced-PACC, especially the parsimonious PACC3_PN, demonstrated the steepest decline—6.4–19% faster than the traditional and animal fluency-appended versions. Our optimized measure offers critical advantages in clinical utility: an 82% reduction in scoring burden for clinicians (9-point proper names versus 50-point total LM score), finer semantic memory granularity within an episodic memory task and enhanced sensitivity to AD biomarkers. Our findings position proper name-enhanced PACC as a clinically practical tool for tracking longitudinal cognitive trajectories, highlighting proper names delayed recall as a sensitive marker due to AD-type brain changes. Future work will validate its utility for trial enrichment and clinical screening.

## Teaser

He *et al*. report that replacing the logical memory total score in the Preclinical Alzheimer’s Cognitive Composite (PACC) with a proper-name subscore yields 6.4–19% faster decline than traditional and animal fluency–appended PACC versions. The proper-name–enhanced PACC improves sensitivity to plasma p-tau217 and reduces clinician scoring burden by 82%.

## Graphical Abstract

**Graphical Abstract:**
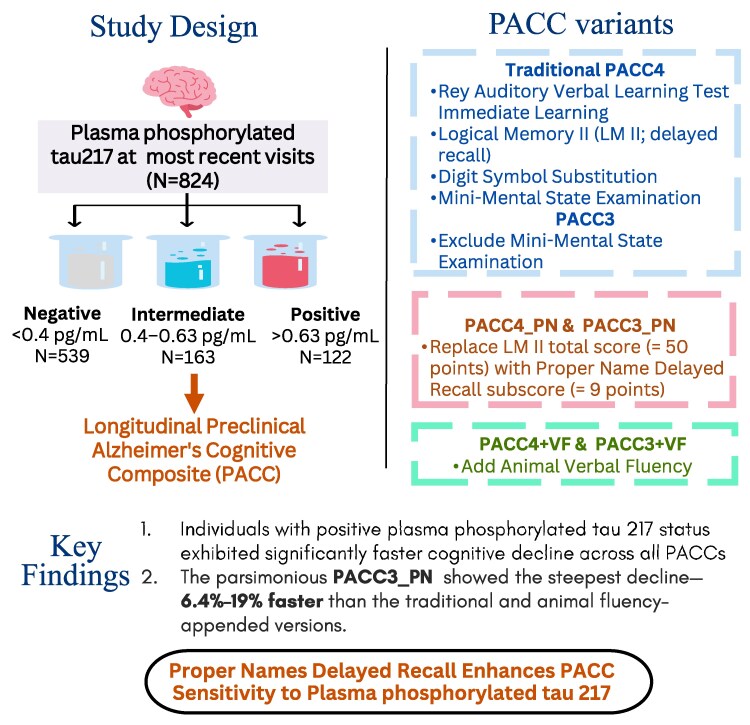

## Introduction

The recent NIA-AA guidelines established a biological definition of Alzheimer’s disease (AD)—abnormal protein deposits alone in the brain are sufficient to define AD, independent of clinical symptoms.^1,2^ Among emerging biomarkers, plasma phosphorylated tau 217 (p-tau217) has shown promise as a non-invasive, cost-effective alternative to PET imaging, with standalone diagnostic value comparable to the established amyloid beta (Aβ) proteinopathy pathway.^2^ However, in the clinical settings, reliance on biomarkers alone raises concerns about overdiagnosis and obscuring the line between risk and definitive diagnosis, particularly among individuals who exhibit pathological changes but never develop cognitive impairment.^3,4^ Therefore, it is imperative to identify sensitive behavioural-cognitive signs that are related to biological changes and demonstrate clinically meaningful therapeutic benefit.

One effort to address this need is the development of cognitive composites such as the Preclinical Alzheimer’s Cognitive Composite (PACC),^5,6^ first introduced by Donohue *et al*. (2014). PACC has served as an outcome measure for the A4 clinical trial, which targeted individuals at the asymptomatic phase of AD to test anti-amyloid treatment.^7^ The original PACC primarily emphasizes tests of episodic memory, executive function and global cognition, where a measure specific for the language domain is lacking. This is a notable gap potentially limiting its sensitivity, given growing evidence showing that language impairments may be among the earliest manifestations of AD.^8-12^ Thus, we hypothesized that the involvement of a language-specific measure to the PACC would improve its sensitivity for early detection. Supporting this, previous studies showed language measure additions to the PACC, such as adding category verbal fluency, that tap semantic processing, improve sensitivity and explain additional variance in amyloid-β related decline.^13,14^

Proper name recall, another promising yet underexplored cognitive-linguistic marker, may offer even greater discriminative power in detecting early AD pathology. Indeed, forgotten proper names of specific persons or places (e.g. ‘Joe’, ‘Mississippi’) are among the most common complaints in older adults^15^ and represent an absolute retrieval challenge for individuals with AD.^11,16-18^ Our lab assesses proper names as a novel item-level measure derived from the delayed story recall subtest, Logical Memory II (LM II), from the Wechsler Memory Scale.^19^ This approach represents a process-based cognitive metric, as it quantifies a specific error type during the test rather than relying solely on global total scores.^20,21^ Notably, baseline proper name delayed recall—but not verbs, numerical expressions or even LM II total score—demonstrated cross-sectional association with amyloid-PET deposition.^22^ Each additional proper name recalled was associated with a 27% reduction in the likelihood of AD biomarker positivity (CSF Aβ_42/40+_/pTau_181+_), sharply contrasting with an 8% reduction observed with LM total scores.^18^ Longitudinal analyses further indicated that baseline proper name delayed recall predicted biomarker status at a mean follow-up of 15.5 years.^23^ Cognitively unimpaired individuals who were biomarker-positive for amyloid-β or tau, measured via PET,^22^ CSF,^11,18^ or plasma assays,^17^ exhibited faster decline in proper names compared to biomarker-negative individuals. These findings suggest that incorporating proper name delayed recall into cognitive composites like the PACC could enhance sensitivity to AD pathology and support the development of clinical-biological consistent models that better reflect the disease process.

Notably, proper name delayed recall is embedded within the standard LM II assessment, widely used in both research and clinical settings. However, it offers greater practical utility over the full LM II (scored 0–50) with its concise scoring range (0–9). Furthermore, proper-name scoring is more objective because a name is either stated correctly or marked incorrect. In contrast, many other idea units scored in LM allow alternative responses (e.g. ‘told the police’ for ‘reported’), which may introduce subjective interpretation and increases the risk of inconsistent scoring. To prioritize clinical applicability and test efficiency, the present study aimed to evaluate whether replacing total LM II score with the proper name delayed recall subscore in the PACC would improve its sensitivity to a diagnostic AD biomarker of plasma p-tau217. We also examined whether a proper name–enhanced PACC performs comparably or better than existing PACC versions incorporating category verbal fluency. Such refinements could advance research and clinical efforts to track disease progression, guide preventive interventions and delay symptom onset.

## Materials and methods

This observational study is reported in accordance with the STROBE guidelines.

### Participants

This study used longitudinal data from Wisconsin Registry for Alzheimer’s Prevention (WRAP), an AD risk-enriched cohort of cognitively unimpaired, late-middle-aged adults with a parental family history of AD. Participants underwent biennial follow-up visits, including neuropsychological testing, lifestyle assessments and blood collection for plasma biomarkers. Cognitive status was determined using internally validated robust norms (derived from longitudinally confirmed cognitively normal individuals)^24-26^ and reviewed by a multidisciplinary consensus team. For each study visit, one of the following cognitive statuses was assigned^26^: cognitively unimpaired-stable (CUS), cognitively unimpaired-declining (CUD; subtle cognitive changes that suggest progression towards MCI or dementia but do not yet meet clinical diagnostic thresholds), MCI (according to NIA-AA criteria^27^), dementia and impaired-not-MCI (i.e. impairments such as due to learning disabilities or longstanding brain dysfunction). Additional methodological details are available elsewhere.^26^

The current analysis used the identical participant sample as our parent study (which focused on the development of language-specific composite scores^28^). They were drawn from the November 2023 WRAP data freeze (*N* = 1751). Inclusion required ≥2 visits of longitudinal cognitive assessments with language measures and concurrent plasma biomarker data. We excluded participants with only a single neuropsychological visit, impaired not-MCI and major neurological conditions (stroke, Parkinson's disease, epilepsy, multiple sclerosis). The final analytic sample comprised 824 baseline dementia-free participants.

Table 1 summarizes participants’ baseline characteristics. Overall, the mean baseline age was ∼62 years, and 67.8% of participants were female. The majority completed three cognitive testing visits over time (median = 3). Primary languages were English (97.5%), Spanish (0.7%), other languages (1.2%) or unspecified (0.6%). Cognitive status at baseline PACC included 661 CUS, 152 CUD and 11 MCI.

**Table 1:** Baseline demographic and clinical characteristics of participants, stratified by most-recent plasma p-tau217 status

	Overall	*Plasma p-tau217 Negative	*Plasma p-tau217 Intermediate	*Plasma p-tau217 Positive	*P*
*N*	824	539	163	122	
Consensus cognitive diagnosis (%)					0.193
Cognitively unimpaired-stable	661 (80.2)	443 (82.19)	128(78.53)	90(73.77)	
Cognitively unimpaired-declining	152 (18.4)	88(16.33)	34(20.86)	30(24.59)	
Clinical MCI	11 (1.3)	8(1.48)	1(0.61)	2(1.64)	
Analysed visits counts, median	3	3	3	3	
Age in years, mean (SD)	61.94 (6.50)	60.99 (6.58)	62.73(5.95)	65.12(5.66)	<0.001
Gender, % Female		68.09	63.8	72.13	0.323
≥1 *APOE*-ε4 allele (%)	313 (38.0)	163(30.24)	74(45.4)	76(62.3)	<0.001
WRAT-III, mean(SD)	106.10 (9.44)	106.05(9.59)	105.29(9.63)	107.41(8.35)	0.167
Cognitive measures (z score)					
Rey AVLT	−0.28 (1.15)	−0.22 (1.14)	−0.39 (1.12)	−0.38 (1.20)	0.145
					
WMS-R Logical Memory-II	−0.13 (1.05)	−0.20 (1.12)	−0.26 (1.20)	−0.38 (1.10)	0.254
WAIS-R Digit Symbol	−0.13 (1.05)	−0.03 (1.06)	−0.23 (1.05)	−0.47 (0.92)	<0.001
Mini-Mental State Exam	−0.11 (1.15)	−0.12 (1.17)	−0.06 (1.14)	−0.12 (1.08)	0.842
Language variables (z-score)					
Proper Name Delayed Recall	−0.18 (1.07)	−0.15 (1.05)	−0.19 (1.07)	−0.30 (1.12)	0.386
Animal Category Fluency	−0.11 (1.00)	−0.06 (0.98)	−0.17 (1.05)	−0.24 (1.01)	0.144

* Following Ashton *et al*. (2024) cut-offs identified via ROC analyses of amyloid PET status, plasma p-tau217 Negative: < 0.40 pg/mL; Intermediate: 0.40–0.63 pg/mL and Positive: > 0.63 pg/mL based on their most-recent visit. Z-scores were constructed using the mean and standard deviation derived from baseline PACC visits for participants who are cognitively unimpaired stable. Descriptive statistics and between-group comparisons were generated using the ‘tableone’ package in R, in which a chi-square test of independence was used for categorical variables and a *t*-test for continuous variables. Consensus Cognitive Diagnosis = cognitive status determined by expert review of longitudinal testing, lifestyle, health and informant report questionaries^24,26^. MCI = Mild Cognitive Impairment; WRAT-III = Wide Range Achievement Test-III Reading Subtest; AVLT = Rey Auditory Verbal Learning Test; WMS-R = Wechsler Memory Scale-Revised; WAIS-R = Wechsler Adult Intelligence Scale-Revised.

Using Ashton *et al*. (2024) cut-offs identified via ROC analyses of amyloid PET status, participants were grouped based on their most recent plasma p-tau217 levels: *negative* (<0.40 pg/mL; *n* = 539), *intermediate* (0.4–0.63 pg/mL; *n* = 163) and *positive* (>0.63 pg/mL; *n* = 122). The mean interval between participants’ baseline and most recent visit was 4.61 years. Compared to the other groups, at the baseline, the p-tau217-positive group was significantly older, had more *APOE* ε4 carriers and showed lower Wechsler Adult Intelligence Scale-Revised (WAIS-R) Digit Symbol scores (all *P* < 0.05; TableOne package, R).

The study protocols were approved by the University of Wisconsin Health Sciences Institutional Review Board. All participants provided informed consent, and procedures adhered to the Helsinki Declaration.

### Language tasks and measures

The Logical Memory (LM) from Wechsler Memory Scale-Revised^19^ is a story recall task that comprises two narratives (stories ‘A’ and ‘B’), each containing 25 distinct ‘idea units’ (e.g. ‘*Anna’, ‘in a school’, ‘the rent was due’*). Participants recalled each story immediately after presentation (LM I = immediate recall) and again after a 20–30 min delay (LM II = delayed recall). Traditionally, the total score sums the number of correctly recalled idea units across both stories, with a maximum of 50 points each for LM I and LM II.

In this study, we stressed a novel item-level process score derived from LM: proper name delayed recall. Proper names constitute a unique lexical category—names of people and places (e.g. *Joe, Mississippi)—*that is particularly vulnerable to individuals with preclinical AD.^17,18,22^ The LM stories contain nine proper names (four in Story A and five in Story B), offering a substantially more concise scoring metric compared to the traditional 50-point total score. We substituted the conventional total score with this proper name subscore to enhance the sensitivity of the PACC framework for early detection.

Further, we benchmarked our proper-name PACC variant against an existing language-augmented PACC5, which has demonstrated enhanced sensitivity to amyloid burden compared to the original PACC.^13^ This PACC5 supplements the core composite with category verbal fluency (i.e. animals, fruits and vegetables)—a widely used semantic memory measure. Therefore, we constructed a parallel version of PACC5 using only animal category fluency as this was the sole category collected in the WRAP cohort. In the animal fluency task, participants were instructed to generate as many unique animal names as possible within 60 s, with total correct responses recorded.

### Composite construction

Following Donohue *et al*. (2014), the WRAP cohort published a modified PACC tailored to its available measures,^30^ including Rey Auditory Verbal Learning Test (AVLT0-75),^31^ LM II (i.e. total score of delayed story recall = 0–50), the Digit Symbol subtest (DS = 0–93) of the WAIS-R^32^ and Mini-Mental State Examination (MMSE = 0–30).^33^ While the original PACC by Donohue *et al*. (2014) used the total score from the Free and Cued Selective Reminding Test (FCSRT; 0–48), WRAP-PACC constructed by Jonaitis *et al*. (2019) substituted AVLT (0–75). Furthermore, the WRAP-PACC combined both stories A and B from LM II for a total score of 0–50 points, whereas Donohue *et al*. (2014) included only story A (0–25 points).

Using the current study sample, we then constructed six PACC variants (Table 2). Variant 1: ‘*PACC4’* is the WRAP-based PACC most closely aligned to Donohue *et al.* (2014)`s PACC.^5,34^ It included AVLT, LM II, DS and MMSE. Variant 2: ‘*PACC3’* excluded MMSE to address its known ceiling effects in cognitively unimpaired adults.^14,35^ Variants 3 and 4: ‘*PACC4_PN’* and ‘*PACC3_PN’* replaced LM II’s total score with the proper name delayed recall subscore, respectively. Variants 5 and 6: ‘*PACC4*  *+*  *VF’* and ‘*PACC3*  *+*  *VF’* were created by the addition of animal category fluency to variants 1 and 2, respectively.

**Table 2:** Indicator selection and composite construction

Indicators		*PACC4	*PACC4_PN	*PACC3	*PACC3_PN	*PACC4 + VF	*PACC3 + VF
Cognitive measures	Rey AVLT Immediate Learning	X	X	X	X	X	X
	WMS-R Logical Memory-II	X		X		X	X
	WAIS-R Digit Symbol Substitution	X	X	X	X	X	X
	MMSE	X	X			X	
Language variables	**Proper Name Delayed Recall		X		X		
	Animal Category Fluency					X	X

*PACC = Preclinical Alzheimer’s Cognitive Composite; PACC4 comprises four cognitive tests, while PACC3 excludes the MMSE due to potential ceiling effects observed in cognitively unimpaired participants; PACC#_PN = Replacing the Logical Memory total score with proper name delayed recall subscore in PACC; PACC#+VF = Adding animal category fluency into PACC; Rey AVLT = Rey Auditory Verbal Learning Test; WMS-R = Wechsler Memory Scale-Revised; WAIS-R = Wechsler Adult Intelligence Scale-Revised; MMSE = Mini-Mental State Exam. X in a cell indicates that the test represented in that row contributed to that column’s composite.

**Note: Proper name delayed recall (range: 0–9) is a subscore from the WMS-R Logical Memory (LM) II test (range:0–50). In other words, all PACC variants include proper name recall either as part of LM II total or as the subscore.

All composite scores were calculated by first standardizing individual test scores into z-scores using the baseline mean and standard deviation (SD) from a cognitively unimpaired-stable^26^ reference subgroup (*n* = 661; Details on demographic characteristics for this subgroup are available in the parent study^28^). Final composites were computed as the unweighted sum of these z-scores, with the aggregate standardized against the mean and SD of all participants and visits to ensure longitudinal consistency and comparability.

### Plasma biomarkers

Plasma samples were collected during the same study visits as cognitive assessments, using EDTA anticoagulants. Analyses were conducted at the Department of Psychiatry and Neurochemistry, University of Gothenburg. The plasma p-tau217 levels were measured via the commercial ALZpath assay that employs a proprietary monoclonal p-tau217-specific capture antibody, an N-terminal detector antibody and a peptide calibrator. This assay demonstrates high predictive accuracy for PET amyloid-β positivity (AUC of 0.93; 95% CI 0.90–0.97) in the WRAP cohort.^29^ Full details are published elsewhere.^29^

### Statistical analysis

We performed all analysis using R (v4.4.0). Descriptive statistics and between-group comparisons in Table 1 were generated using the ‘tableone’ package in R, in which a chi-square test of independence was used for categorical variables and a *t*-test for continuous variables.

We examined longitudinal trajectories of cognitive decline (i.e. PACCs) in relation to the most-recent plasma p-tau 217 levels using linear mixed-effects models (lme4 package, R). The primary model was *PACCs* ∼ *p-tau217 group × centered_age + WRAT-III + sex + practice,* with subject-specific intercepts as random effects. Age was centred at 60 years and used as the time metric. To account for practice effects often observed in LM,^23,36^ we calculated ‘practice’ as the number of prior test exposures (total visit number) minus one.^37,38^ The p-tau217 group was modelled as a fixed effect with three levels (negative, intermediate and positive). Literacy (Wide Range Achievement Test-III; WRAT-III), sex and practice were included as covariates, and participants’ IDs were the random intercepts to account for within-subject correlations over time.

Residual diagnostic plots indicated that the linear mixed-effects model assumptions were largely met for all analysis, including the PACC variants using proper name subscore despite its limited score range. Sensitivity to plasma p-tau217 was compared across PACC variants utilizing β estimates for the p-tau 217-positive group * centred age interaction, with significance set at *P* < 0.05 (95% CIs reported). Statistical significance (β/SE) was expressed as beta coefficients divided by standard error.

## Results

**Figure 1:**
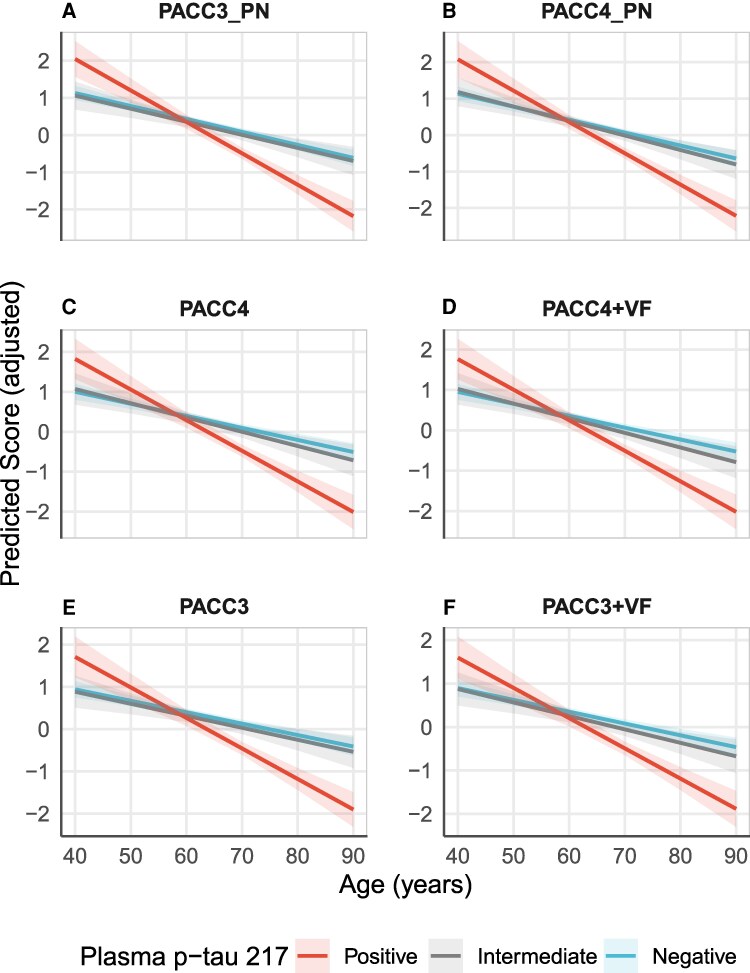
**Longitudinal decline (i.e. predicted trajectories) in cognitive composites by plasma p-tau217 status from linear mixed effects models**. The figures display the β estimates of PACCs in descending order from A to F. Individuals with positive plasma p-tau217 (*n* = 122) showed a faster decline rate compared to those negative (*n* = 539) across all composites. The PACC incorporating proper name (i.e. PACC_PN; panels A and B) demonstrated the steepest decline rate compared to original PACC (using Logical Memory total score; panels C and E) and the PACC appended with animal verbal fluency (panels D and F). Each panel depicts the predicted age trajectory derived from the linear mixed models of ‘composites z-score ∼ p-tau217 group * centred age + literacy + sex + practice’ plus random effects for subject-specific intercepts. Age was centred at 60 and practice was calculated as total visits number minus one. Age-related slopes differed significantly (*P* < 0.001) for plasma p-tau217-positive versus -negative groups but not between intermediate and negative groups (*P* > 0.05) for all composites. PACC#= Preclinical Alzheimer’s Cognitive Composite (default with Logical Memory II total score). PACC#_PN: PACC with Logical Memory II total score replaced by proper name delayed recall subscore; PACC#+VF: PACC supplemented with animal fluency.

Our linear mixed effects models showed significant plasma p-tau217 group * centred age interactions across all PACC variants (all *P* < 0.05; Fig. 1; Supplementary Table 1), suggesting accelerated rates of cognitive decline in positive p-tau217 individuals. Age, literacy, sex and practice emerged as significant covariates (all *P* < 0.05), with older age predicting lower cognitive composite scores, while female sex, higher literacy and more practice were associated with better performance.

Although the PACC variants appear visually similar in Fig. 1, this similarity itself is informative: all variants showed strong sensitivity to longitudinal change associated with plasma p-tau217, consistent with prior work evaluating PACC variants.^5,13,34^ To enable sensitivity comparison, Table 3 ranks variables by the magnitude of β coefficients for p-tau217-positive * centred age interaction. The PACC3_PN (β = −0.050; 95% CI, −0.068 to −0.031), and PACC4_PN (β = −0.050; 95% CI, −0.070 to −0.031) demonstrated the strongest association with p-tau217–related decline, followed by PACC4 (β = −0.047; 95% CI, −0.066 to −0.027), PACC4 + VF (β = −0.046; 95% CI, −0.065 to −0.027), PACC3 (β = −0.045; 95% CI, −0.063 to −0.027) and PACC3 + VF (β = −0.042; 95% CI, −0.061 to −0.024).

**Table 3:** Longitudinal decline rate comparison across composites

Composites^a^	β estimates^b^	β SE	Statistical significance^c^	*P*	95% CI	Decline rate comparison^d^
PACC3_PN	−0.050	0.0093	−5.376	< 0.001	[−0.068, −0.031]	reference (fastest)
PACC4_PN	−0.050	0.0098	−5.102	< 0.001	[−0.070, −0.031]	No difference (Δ0%)
PACC4	−0.047	0.0098	−4.796	< 0.001	[−0.066, −0.027]	↓6.4%
PACC4 + VF	−0.046	0.0098	−4.694	< 0.001	[−0.065, −0.027]	↓8.7%
PACC3	−0.045	0.0093	−4.839	< 0.001	[−0.063, −0.027]	↓11%
PACC3 + VF	−0.042	0.0094	−4.468	< 0.001	[−0.061, −0.024]	↓19%

^a^PACC = Preclinical Alzheimer’s Cognitive Composite; PACC4 comprises four core tests, while PACC3 excludes the MMSE due to potential ceiling effects observed in cognitively unimpaired participants; PACC#_PN = Replacing the Logical Memory total score with proper name delayed recall subscore in PACC; PACC#+VF = Adding animal category fluency into PACC.

^b^The β estimates were ranked by magnitude (steepest to flattest age-related decline) to compare their sensitivity to plasma biomarker across PACC variants. They were derived from the interaction effects of p-tau 217-positive group *centred age from linear mixed models adjusting for age, gender, literacy and practice effects. The *p* value indicates the significance of this interaction.

^c^Statistical significance = β/SE; the estimate (β) for p-tau 217-positive group *centred age divided by β SE, i.e. the simple age slope for the plasma p-tau217-positive group divided by the standard error.

^d^The comparison of decline rates (i.e. β estimates) was calculated between the fastest decline on PACC3_PN and other variants.

Notably, among individuals with positive p-tau217, both the PACC3_PN and PACC4_PN presented the fastest rate of cognitive decline—6.4 to 19.0% faster—than the original and verbal fluency-appended PACC variants.

Following previous studies using β/SE to compare PACC variants,^13,39^ PACC3_PN (β/SE = −5.376) showed the strongest statistical significance, followed by PACC4_PN (β/SE = −5.102). Both outperformed the original (PACC3: β/SE = −4.839; PACC4: β/SE = −4.796) and verbal fluency-appended PACCs (PACC4 + VF: β/SE = −4.694; PACC3 + VF: β/SE = − 4.468).

## Discussion

This retrospective cohort study evaluated the performance of PACC variants in tracking longitudinal cognitive decline in relation to plasma p-tau217, a standalone diagnostic biomarker for AD.^1,2^ We hypothesized that composites incorporating proper name delayed recall—a process-based measure targeting the AD-vulnerable domain of semantic memory—would better capture p-tau217-associated decline. To prioritize clinical feasibility, we introduced novel PACC_PN composites by replacing the 50-point LM total score with its subscore, 9-point proper names. We found that (1) individuals with positive plasma p-tau217 experienced accelerated cognitive decline across all PACC variants and (2) the proper name-enhanced PACC, especially the most parsimonious PACC3_PN, showed the strongest association with AD pathological changes, capturing faster cognitive decline, 6.4–19.0% greater than the original and verbal fluency–appended PACC variants. This optimized measure offers critical advantages in clinical utility: reduced scoring burden for clinicians, finer granularity in assessing lexical-semantic memory in the context of an episodic memory task and enhanced sensitivity to subtle cognitive decline for biologically positive AD individuals. In our WRAP samples, findings position PACC_PN as an optimal and clinically practical tool for detecting cognitive decline and highlight the enhanced sensitivity of proper name delayed recall associated with AD-related neuropathology.

The emergence of plasma p-tau217 as a robust standalone diagnostic biomarker for AD marks a significant advance in detecting early pathological changes. In this context, we showed that individuals with positive p-tau217 exhibited faster longitudinal decline across all PACC variants compared to those who were p-tau217-negative, reinforcing the utility of multidomain cognitive composites as sensitive measures reflecting early AD pathological changes. A key innovation of our study was replacing LM total score with a proper name subscore—a process-based, lexical-specific measure from an episodic memory test—yielding enhanced PACC_PN that outperformed conventional PACC4 and PACC3 by 6.4% and 11%, respectively. This result indicates that language-modified PACCs may improve sensitivity to AD pathology-related cognitive decline, corroborated by prior findings.^12-14^ The subtle suboptimal performance of the original PACC likely reflects its constrained sensitivity to language-mediated cognitive decline that is evident in early AD, as over half its weight is placed on episodic memory (i.e. FCSRT or AVLT, LM and partial MMSE),^34^ despite equal component weighting. Our findings suggest that the inclusion of language measures is important for optimizing composite endpoints as they can capture the earliest cognitive manifestations of AD pathology.^8-12^

However, unlike prior findings,^13,14^ we found the added benefit was derived specifically from proper name delayed recall rather than category verbal fluency. Compared to the standard PACC4, neither longitudinal decline rate nor statistical tests of differences (β/SE) showed improvements for verbal fluency-appended PACCs in our WRAP cohort. Instead, relative to PACC4 + VF and PACC3 + VF, the proper name–enhanced PACC was more sensitive to longitudinal change, capturing 8.7% and 19% greater cognitive decline, respectively, as indicated by the model-estimated β slopes. Although both verbal fluency and proper name recall draw on semantic memory, we posit fundamental distinctions rooted in semantic network processing underlie these findings. Proper names differ substantially from common nouns retrieved in category fluency: they are unique, often arbitrary, lack synonyms and are sparsely connected within semantic networks,^40,41^ whereas common semantic or conceptual categories (e.g. animals, fruits) feature more robustly organized semantic networks and are readily supported by clustering or associative activations.^41,42^ In addition, proper names were retrieved in a narrative (connected speech), which added further cognitive load compared to the isolated, single-word responses in category fluency tasks. Alternatively, this discrepancy could be simply due to our WRAP cohort’s use of a single-category animal fluency task, whereas Papp *et al*. (2017) suggested multi-category fluency tasks to maximize amyloid +/− group differentiation in the Harvard Aging Brain Study.^13^ Indeed, slight variations in PACCs performance are expected across cohorts due to differences in measures and population characteristics.^43^ Regardless, these findings strengthen the growing evidence that language deficits, particularly those tapping semantic memory from connected speech, are important detectable signs in early AD.^8-12^

Delayed proper name recall performed comparably, if not better, than the LM total score in both longitudinal and cross-sectional findings.^17,22,44^ Our study extends these findings by demonstrating that, when incorporated into the PACC, a focused 9-item proper name recall outperformed the 50-point LM total score in distinguishing plasma p-tau217-positive individuals. This result was further strengthened by an independent BIOCARD cohort, where even a 4-item proper name score from Story A alone matched sensitivity to the full story when identifying positive amyloid status from cognitively unimpaired individuals.^23^ These notable sensitivities are particularly striking given that the LM total score itself comprises and extends beyond proper names. All PACC variants, thereby, contain proper names either alongside other idea units or concisely as a self-sufficient measure. However, enhanced sensitivity was observed with a parsimonious PACC that includes specificity for retrieving names of people and places, suggesting that the traditional LM total score may obscure such deficits due to score aggregation. Altogether, these findings suggest that proper names, whether as a targeted measure or part of aggregated scores, are sensitive and efficient markers for early AD detection but with particular clinical value when assessed as a focused measure.

Indeed, our proper name enhanced-PACC presents clear practical advantages. It improves clinical feasibility by an 82% reduction in scoring burden (9-point versus 50-point scale), while enhancing pathological specificity through its exclusive probing on the AD-vulnerable semantic network. Furthermore, its lack of synonymous alternatives minimizes scoring variability and maximizes raters′ reliability, making it well-suited for use in high-demand clinical settings. Given that proper name recall is an existing subscore of the LM test, this optimized PACC_PN can be seamlessly applied to both retrospective datasets with item-level entries and future studies, streamlining its adoption in research and routine practice. The parsimonious PACC3_PN, in particular, provides the best feasible alternative to the original PACC4 that could facilitate earlier and more accessible identification of at-risk individuals. Moreover, future research may benefit from considering proper name recall in both experimental paradigms and the development of new cognitive assessments.

While the observed β differences between composites remain clinically relevant, we acknowledge that the overlapped confidence intervals for β estimates limit our generalizability beyond the WRAP cohort. Future studies should validate our findings in independent, demographically diverse populations and/or employ data harmonization methods to facilitate direct comparisons between PACC variants. While we expect our main conclusions to be broadly consistent under comparable specifications, comparisons could vary under alternative analytic frameworks (e.g. mixed models for repeated measures, MMRM) and that future work directly comparing these frameworks would help assess robustness. Another future direction is to validate whether composite scores like the PACC outperform its individual components, such as proper name recall and digit symbol. A caveat to consider in applying our optimized PACC_PN to clinical settings is that some constituent measures of the PACC_PN (including the WMS-R/WAIS-R versions of Logical Memory and Digit Symbol Substitution) have been updated by the test publisher; generalizability to alternate forms and stories is therefore an important future direction for determining its clinical utility in contemporary practice.^43^ In addition, PACC variants that do not include a story memory component cannot accommodate substitution of proper-name recall for total story recall.

In conclusion, our findings identify forgotten proper names as sensitive, clinically meaningful cognitive-linguistic markers that significantly enhance the PACC composite’s biological relevance. This approach prioritizes a process score rather than a total score modernizing a classic assessment tool and enhancing the logical memory tests with added value for contemporary research and clinical practice. Our optimized PACC_PN variants may serve as ideal cognitive endpoints for tracking early AD progression when combined with plasma p-tau217, providing a direct solution for improving clinical accessibility and feasibility.

## Supplementary Material

fcag163_Supplementary_Data

## Data Availability

The data that support the findings of this study are available from the Wisconsin Registry for Alzheimer’s Prevention (WRAP). Data are not publicly available but may be obtained by submitting a request directly to WRAP at https://wrap.wisc.edu. The R code used for data analysis in this manuscript is provided in the Supplementary material.
