# Supplementary material for: Enhanced sensitivity to plasma p-tau217 with proper names recall in the preclinical Alzheimer’s cognitive composite
Source: Brain Commun. 2026 May 6;8(3):fcag163. doi: 10.1093/braincomms/fcag163 (PMC13195032; doi:10.1093/braincomms/fcag163)
Supplement: fcag163_Supplementary_Data [file fcag163_supplementary_data.docx]

**Supplemental material**

**eTable 1. Most recent plasma p-tau 217 predicting longitudinal decline of PACC variants using linear mixed models**.

|  | **PACC3_PN** | | | **PACC4_PN** | | | **PACC4** | | |
| --- | --- | --- | --- | --- | --- | --- | --- | --- | --- |
| *Predictors* | *Estimates* | *CI* | *p* | *Estimates* | *CI* | *p* | *Estimates* | *CI* | *p* |
| (Intercept) | -4.096 | -4.731 – -3.460 | **<0.001** | -4.428 | -5.044 – -3.812 | **<0.001** | -4.540 | -5.163 – -3.917 | **<0.001** |
| Group [Intermediate] | -0.075 | -0.229 – 0.078 | 0.337 | -0.036 | -0.188 – 0.115 | 0.637 | -0.041 | -0.194 – 0.112 | 0.599 |
| Group [Positive] | -0.079 | -0.274 – 0.117 | 0.431 | -0.064 | -0.260 – 0.132 | 0.522 | -0.109 | -0.306 – 0.089 | 0.281 |
| Centered age | -0.035 | -0.042 – -0.027 | **<0.001** | -0.035 | -0.043 – -0.028 | **<0.001** | -0.030 | -0.038 – -0.022 | **<0.001** |
| WRAT-III | 0.030 | 0.024 – 0.036 | **<0.001** | 0.033 | 0.028 – 0.039 | **<0.001** | 0.034 | 0.029 – 0.040 | **<0.001** |
| Gender [Female] | 0.634 | 0.522 – 0.747 | **<0.001** | 0.605 | 0.497 – 0.714 | **<0.001** | 0.585 | 0.475 – 0.695 | **<0.001** |
| Practice | 0.167 | 0.111 – 0.224 | **<0.001** | 0.159 | 0.104 – 0.213 | **<0.001** | 0.168 | 0.113 – 0.223 | **<0.001** |
| Group [Intermediate] × Centered age | -0.000 | -0.016 – 0.016 | 0.975 | -0.004 | -0.021 – 0.013 | 0.618 | -0.006 | -0.022 – 0.011 | 0.519 |
| Group [Positive] × Centered age | -0.050 | -0.068 – -0.031 | **<0.001** | -0.050 | -0.070 – -0.031 | **<0.001** | -0.047 | -0.066 – -0.027 | **<0.001** |
| **Random Effects** | | | | | | | | | |
| σ^2^ | 0.19 | | | 0.25 | | | 0.25 | | |
| τ_00_ | 0.51 _ID_ | | | 0.45 _ID_ | | | 0.46 _ID_ | | |
| ICC | 0.73 | | | 0.64 | | | 0.65 | | |
| N | 824 _ID_ | | | 824 _ID_ | | | 824 _ID_ | | |
| Observations | 2167 | | | 2167 | | | 2167 | | |
| Marginal R^2^ / Conditional R^2^ | 0.279 / 0.807 | | | 0.286 / 0.746 | | | 0.271 / 0.747 | | |

|  | **PACC3** | | | **PACC4+VF** | | | **PACC3+VF** | | |
| --- | --- | --- | --- | --- | --- | --- | --- | --- | --- |
| *Predictors* | *Estimates* | *CI* | *p* | *Estimates* | *CI* | *p* | *Estimates* | *CI* | *p* |
| (Intercept) | -4.212 | -4.859 – -3.565 | **<0.001** | -4.587 | -5.224 – -3.950 | **<0.001** | -4.301 | -4.958 – -3.643 | **<0.001** |
| Group [Intermediate] | -0.084 | -0.240 – 0.072 | 0.290 | -0.061 | -0.217 – 0.095 | 0.443 | -0.097 | -0.255 – 0.061 | 0.230 |
| Group [Positive] | -0.136 | -0.334 – 0.062 | 0.179 | -0.111 | -0.311 – 0.089 | 0.275 | -0.148 | -0.349 – 0.053 | 0.148 |
| Centered age | -0.027 | -0.035 – -0.019 | **<0.001** | -0.030 | -0.037 – -0.022 | **<0.001** | -0.027 | -0.035 – -0.020 | **<0.001** |
| WRAT-III | 0.030 | 0.025 – 0.036 | **<0.001** | 0.035 | 0.030 – 0.041 | **<0.001** | 0.032 | 0.027 – 0.038 | **<0.001** |
| Gender [Female] | 0.607 | 0.493 – 0.722 | **<0.001** | 0.472 | 0.360 – 0.585 | **<0.001** | 0.468 | 0.352 – 0.585 | **<0.001** |
| Practice | 0.176 | 0.119 – 0.233 | **<0.001** | 0.164 | 0.107 – 0.220 | **<0.001** | 0.170 | 0.112 – 0.229 | **<0.001** |
| Group [Intermediate] × Centered age | -0.001 | -0.017 – 0.015 | 0.869 | -0.007 | -0.024 – 0.010 | 0.431 | -0.004 | -0.020 – 0.012 | 0.649 |
| Group [Positive] × Centered age | -0.045 | -0.063 – -0.027 | **<0.001** | -0.046 | -0.065 – -0.027 | **<0.001** | -0.042 | -0.061 – -0.024 | **<0.001** |
| **Random Effects** | | | | | | | | | |
| σ^2^ | 0.18 | | | 0.23 | | | 0.18 | | |
| τ_00_ | 0.54 _ID_ | | | 0.50 _ID_ | | | 0.56 _ID_ | | |
| ICC | 0.75 | | | 0.69 | | | 0.75 | | |
| N | 824 _ID_ | | | 824 _ID_ | | | 824 _ID_ | | |
| Observations | 2167 | | | 2167 | | | 2167 | | |
| Marginal R^2^ / Conditional R^2^ | 0.254 / 0.812 | | | 0.249 / 0.764 | | | 0.228 / 0.811 | | |

**R code examples**

data<-data %>%

mutate(c_age= age_at_appointment-60)

data$mean_conc<-as.numeric(as.character(data$mean_conc)) # mean_conc = plasma p-tau217 level

# Categorize mean_conc into 'Positive', 'Negative', and 'UD'

data_grouped <- data %>%

group_by(ID) %>%

slice_max(order_by = VisNo, with_ties = FALSE) %>% # Get the most recent visit for each ID

mutate(

VisRec = VisNo-1, # assign most recent VisNo

group = factor( case_when(

mean_conc >= 0.63 ~ "Positive",

mean_conc <= 0.40 ~ "Negative",

TRUE ~ "UD"

), levels = c("Negative","UD", "Positive" ))) %>%

ungroup()

sample_size <- data_grouped %>%

group_by(group) %>%

summarise(sample_size = n(), .groups = "drop")

print(sample_size)

data <- data %>%

left_join(data_grouped %>% select(ID, group, VisRec), by = "ID")

## z_cog4 = PACC4; z_cog_pro = PACC4_PN; z_cog3= PACC3; z_cog3_pro= PACC3_PN; ##z_cog3_vf= PACC3+VF; z_cog3_vf= PACC4_VF

lm1 <- lmer(z_cog4 ~ group * c_age + readstn +gender + VisRec+ (1 | ID), data = data, REML = FALSE)

resid_panel(lm1,plots = "all") #diagnostics

lm2 <- lmer(z_cog4_pro ~ group * c_age + readstn +gender + VisRec+(1 | ID), data = data, REML = FALSE)

resid_panel(lm2,plots = "all") #diagnostics

lm3 <- lmer(z_cog3 ~ group * c_age + readstn +gender + VisRec+ (1 | ID), data = data, REML = FALSE)

resid_panel(lm3,plots = "all") #diagnostics

lm4 <- lmer(z_cog3_pro ~ group * c_age + readstn +gender + VisRec+(1 | ID), data = data, REML = FALSE)

resid_panel(lm4,plots = "all") #diagnostics

lm5 <- lmer(z_cog3_vf ~ group * c_age + readstn +gender + VisRec+ (1 | ID), data = data, REML = FALSE)

resid_panel(lm5,plots = "all") #diagnostics

lm6 <- lmer(z_cog4_vf ~ group * c_age + readstn +gender + VisRec+ (1 | ID), data = data, REML = FALSE)

resid_panel(lm6,plots = "all") #diagnostics

tab_model(lm1, lm2, lm3, lm4, lm5, lm6, digits=3)

##get predicted values after adjusting for readstn and gender, and practice

# Define models with descriptive names

models <- list(

"PACC3_PN" = lm4,

"PACC4_PN" = lm2,

"PACC4" = lm1,

"PACC4+VF" = lm6,

"PACC3" =lm3,

"PACC3+VF"=lm5

)

# Generate predictions for all models

all_pred <- imap_dfr(models, ~ {

ggpredict(.x,

terms = c("c_age", "group"),

condition = c(

readstn = mean(data$readstn, na.rm = TRUE),

VisRec =mean (data$VisRec, na.rm=TRUE),

gender = 2 # Use your reference level

)

) %>%

as_tibble() %>%

mutate(outcome = .y) # Add model identifier

})

readstn = mean(data$readstn, na.rm = TRUE)

# Convert centered age to original age (+60)

all_pred <- all_pred %>%

mutate(

original_age = x + 60, # Reverse centering transformation

outcome = factor(outcome, levels = names(models)) # Preserve model list order

)

# Journal-inspired color scheme (Colorblind-friendly)

journal_colors <- c(

"Positive" = "#E64B35",

"UD" = "gray50",

"Negative" = "#4DBBD5"

)

# Create combined plot

combined_plot <- ggplot(all_pred, aes(x = original_age, y = predicted, color = group)) +

geom_line(linewidth = 0.8) +

geom_ribbon(

aes(ymin = conf.low, ymax = conf.high, fill = group),

alpha = 0.15,

linetype = "blank"

) +

facet_wrap(~outcome, ncol = 3, scales = "free_y") +

labs(

x = "Age (years)",

y = "Predicted Score (adjusted)",

color = "Group",

fill = "Group"

) +

scale_color_manual(

values = journal_colors,

breaks = c("Positive", "UD", "Negative"),

labels = c(

"Positive" = "Positive",

"UD" = "Intermediate",

"Negative" = "Negative"

),

name = "Plasma p-tau 217"

) +

scale_fill_manual(

values = journal_colors,

breaks = c("Positive", "UD", "Negative"),

labels = c(

"Positive" = "Positive",

"UD" = "Intermediate",

"Negative" = "Negative"

),

name= "Plasma p-tau 217"

) +

scale_x_continuous(

breaks = seq(0, 120, by = 10),

limits = c(min(all_pred$original_age), max(all_pred$original_age))

) +

theme_minimal(base_size = 12) +

theme(

legend.position = "bottom",

strip.text = element_text(face = "bold", size = 10),

panel.spacing = unit(1.5, "lines"),

panel.grid.minor = element_blank(),

panel.border = element_rect(fill = NA, color = "gray80"), # Subtle panel borders

axis.line = element_line(color = "gray30") # Clear axis lines

)

print(combined_plot)
